# Supplementary material for: Novel Gene Signatures Predictive of Patient Recurrence-Free Survival and Castration Resistance in Prostate Cancer
Source: Cancers (Basel). 2021 Feb 22;13(4):917. doi: 10.3390/cancers13040917 (PMC7927111; doi:10.3390/cancers13040917)
Supplement: Supplementary file 1 [file cancers-13-00917-s001.pdf]

cancers

MDPI

Supplementary Materials: Novel Gene Signatures Predictive of Patient Recurrence-Free Survival and Castration Resistance in Prostate Cancer

Jun A, Baotong Zhang, Zhiqian Zhang, Jin-Tang Dong

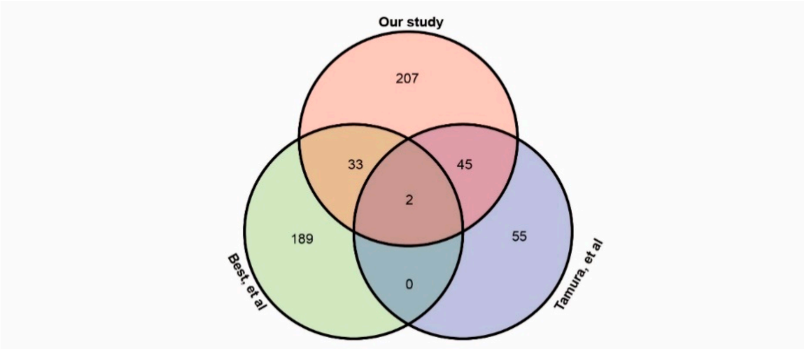

Figure S1. Overlap differentially expressed genes (DEGs) between our study and previously reported microarray experiments.

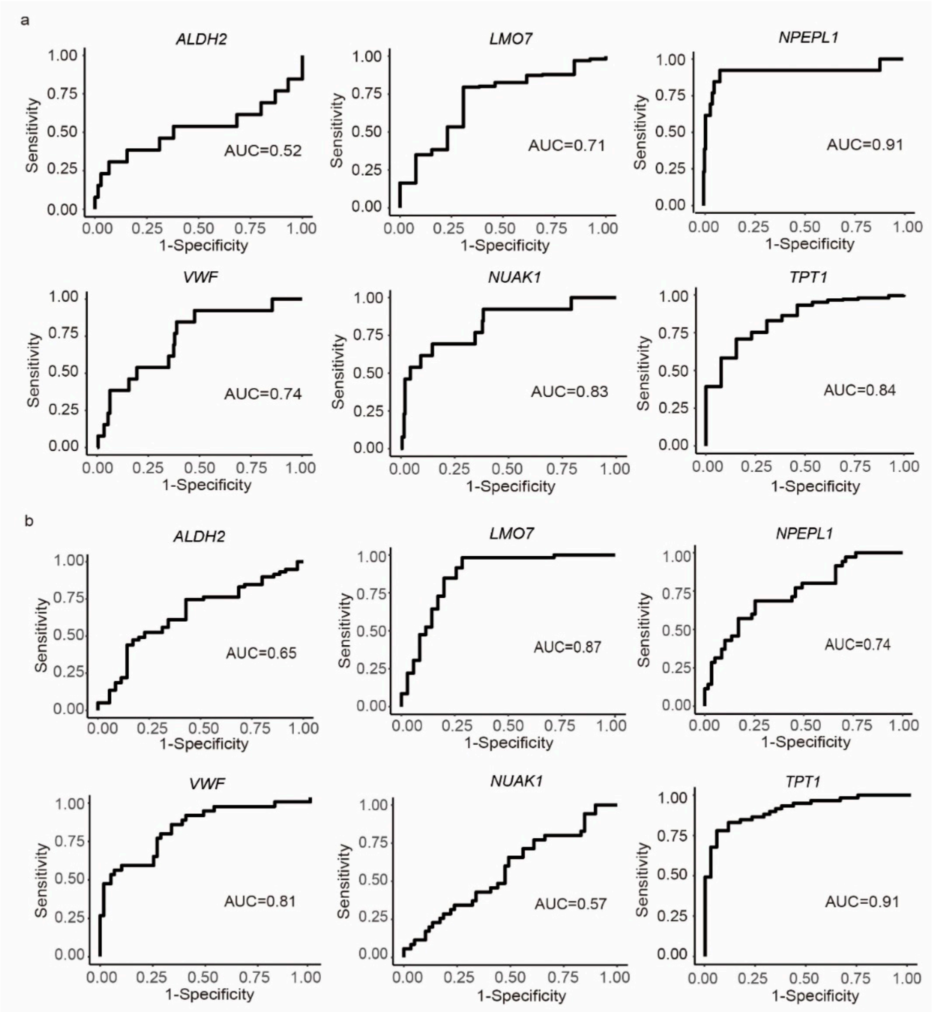

Figure S2. The diagnosis effectiveness of the six CRPCPS genes. ROC curves for the six CRPCPS genes in the training cohort **(a)** and the validation cohort **(b)**.

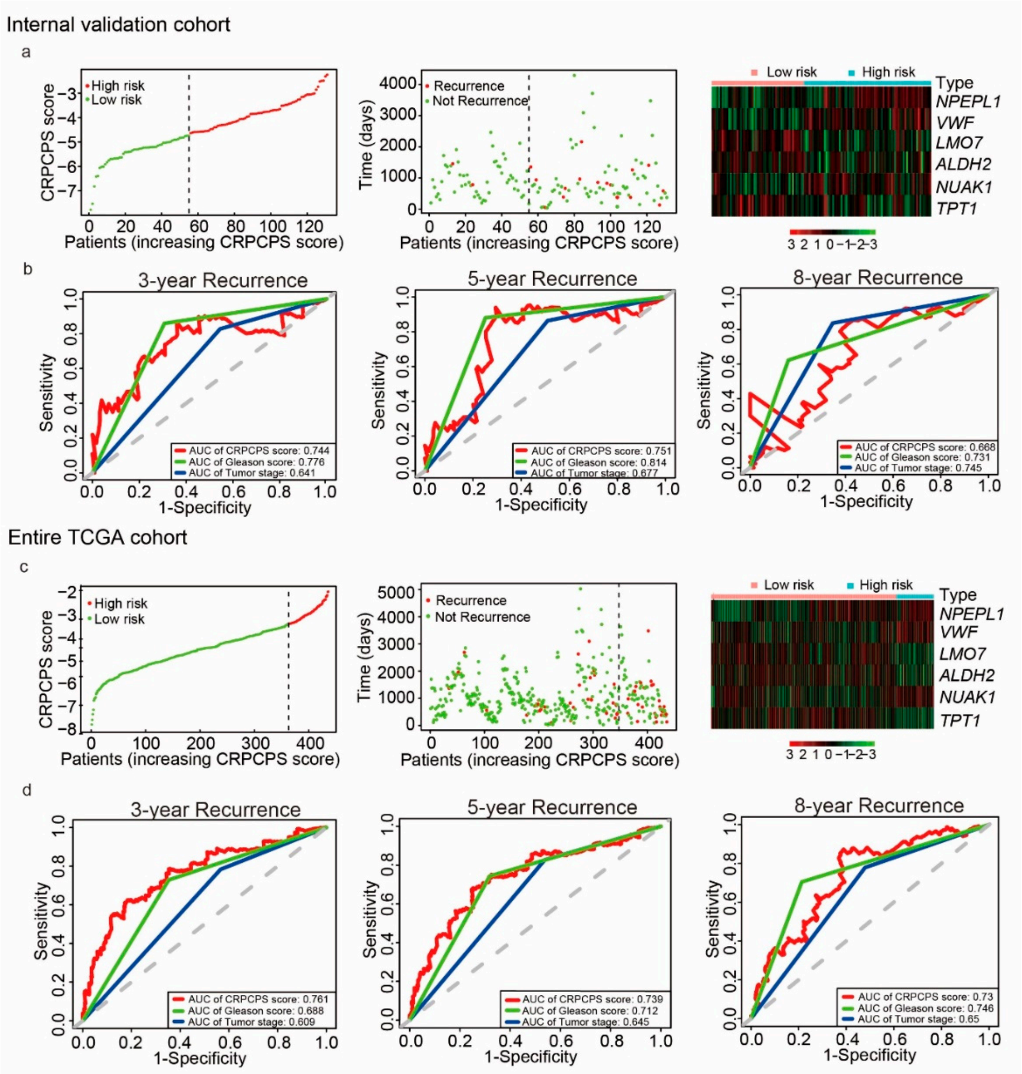

Figure S3. Evaluation of the CRPCPS in the internal validation cohort and the entire TCGA cohort. **(a)** Distribution of CRPCPS score (left), patients' recurrent status (center), and expression profiles of the six CRPCPS genes (right) in the internal validation cohort. **(b)** Receiver operating characteristic (ROC) curves were used to evaluate the predictability of RFS at 3-, 5-, and 8-year by the CRPCPS score, Gleason score, and pathological tumor stage in the internal validation cohort. **(c)** Distribution of CRPCPS score (left), patients' survival status (center), and expression profiles of the six CRPCPS genes that constitute the CRPCPS (right) in the entire TCGA cohort. (**d**) Receiver operating characteristic (ROC) curves were used to evaluate the predictability of RFS at 3-, 5-, and 8-year by the CRPCPS score, Gleason score, and pathological tumor stage in the entire TCGA cohort.

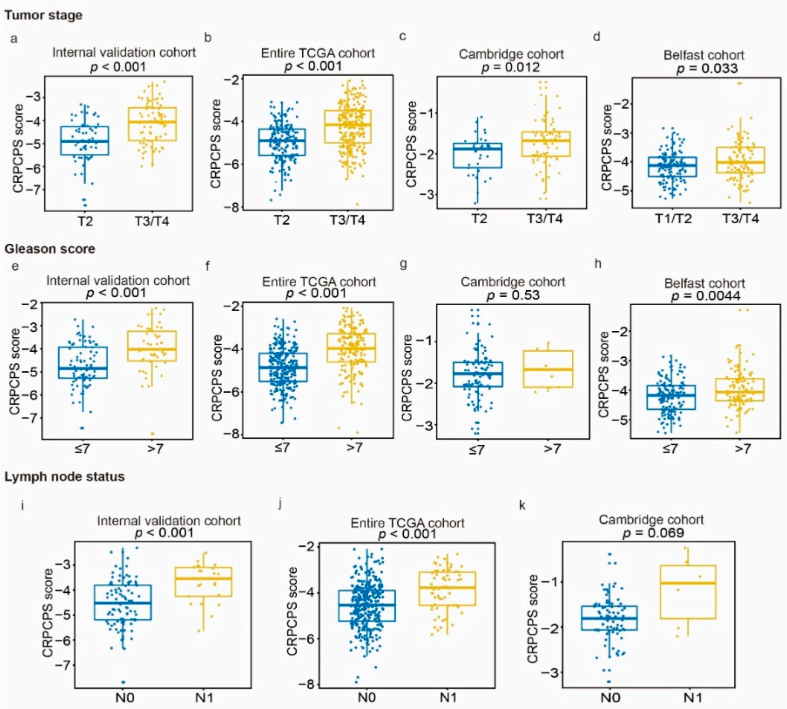

Figure S4. Association of CRPCPS with tumor stage **(a-d)**, Gleason score **(e-h)**, and lymph node status **(i-k)** in different patient cohorts.

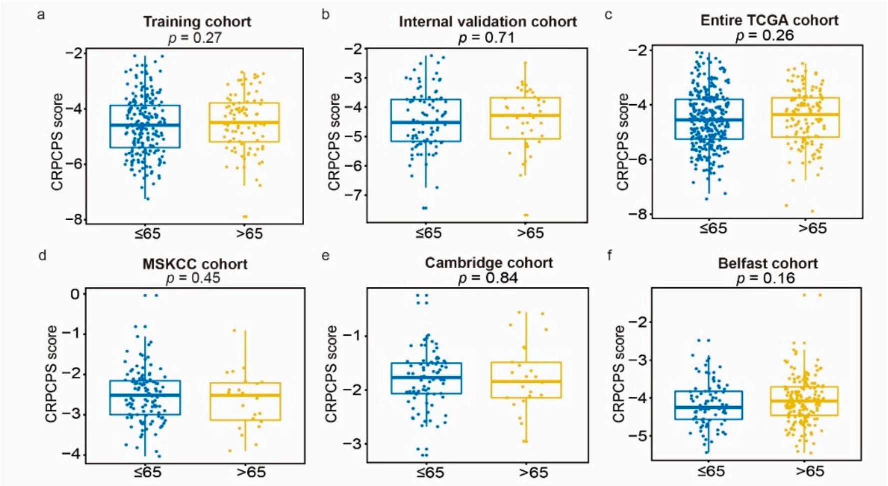

Figure S5. Association of CRPCPS with patients' age in the TCGA training cohort **(a)**, the TCGA validation cohort **(b)**, the entire TCGA cohort **(c),** the MSKCC cohort **(d)**, the Cambridge cohort (GSE70768) **(e)**, and the Belfast cohort (GSE116918) **(f)**.

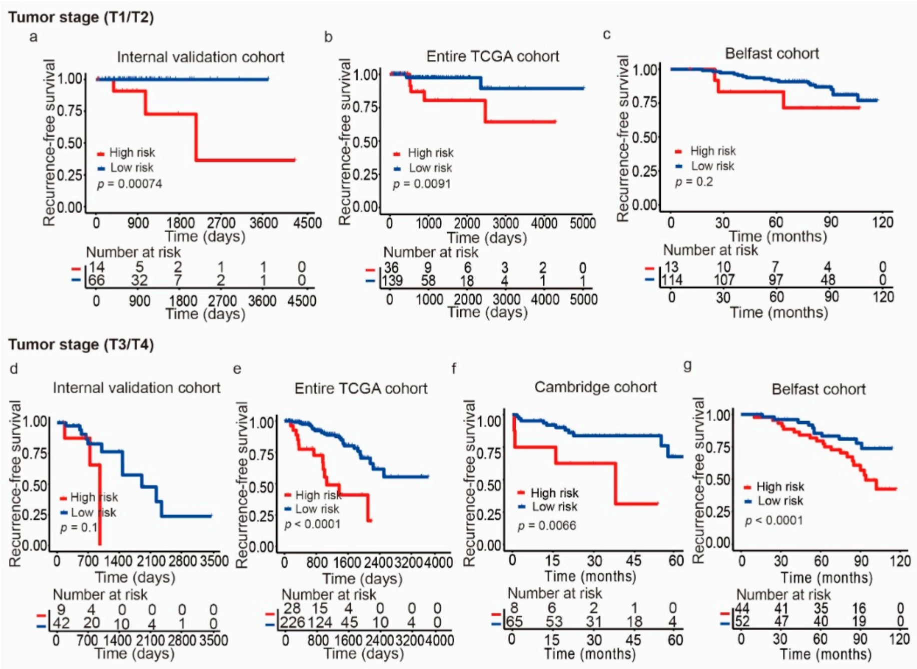

Figure S6. Kaplan-Meier curves of recurrence-free survival (RFS) according to the CRPCPS score in patients with lower (T1/T2) **(a-c)** and higher pathological tumor stage (T3/T4) **(d-g)** in indicated patient cohorts. The tick marks on the Kaplan-Meier curves represent the censored subjects. The two-sided log-rank test was used to determine differences between two curves.

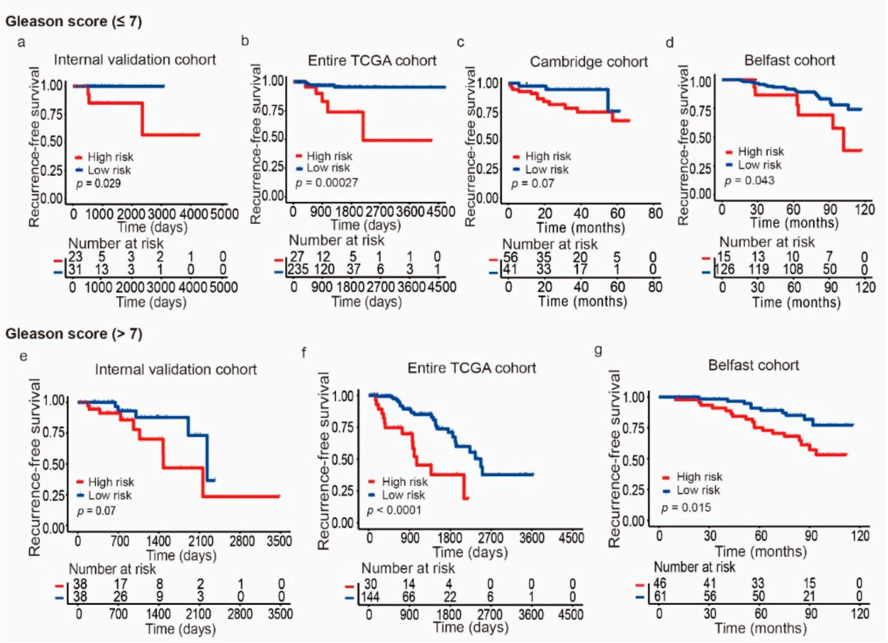

Figure S7. Kaplan-Meier curves of recurrence-free survival (RFS) according to the CRPCPS score in patients with lower (≤ 7) **(a-d)** and higher Gleason score (> 7) **(e-g)** in indicated patient cohorts. The tick marks on the Kaplan-Meier curves represent the censored subjects. The two-sided log-rank test was used to determine differences between two curves.

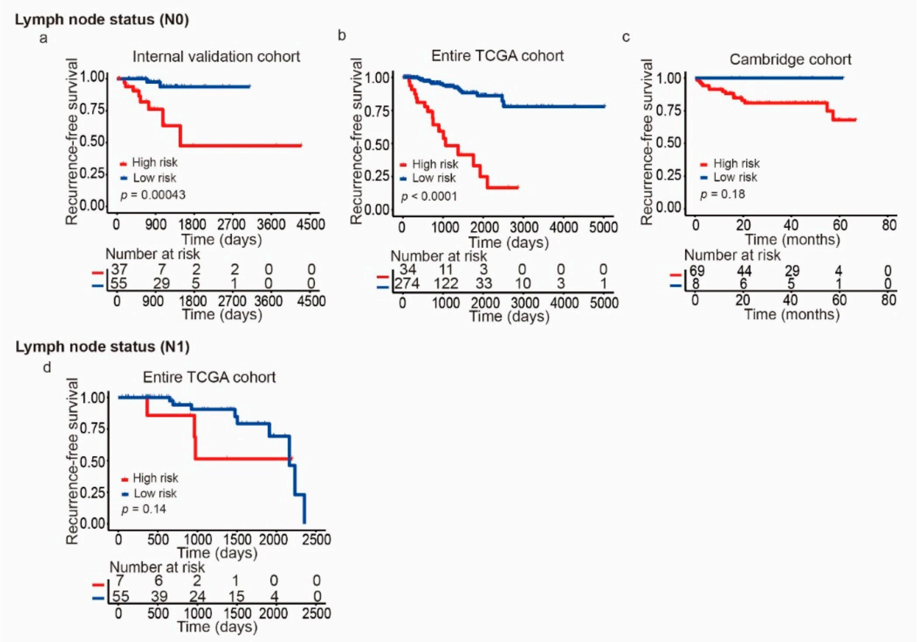

Figure S8. Kaplan-Meier curves of recurrence-free survival (RFS) according to the CRPCPS score in patients without (N0) **(a–c)** and with lymph node metastasis (N1) **(d)** in indicated patient cohorts. The tick marks on the Kaplan-Meier curves represent the censored subjects. The two-sided log-rank test was used to determine differences between two curves.

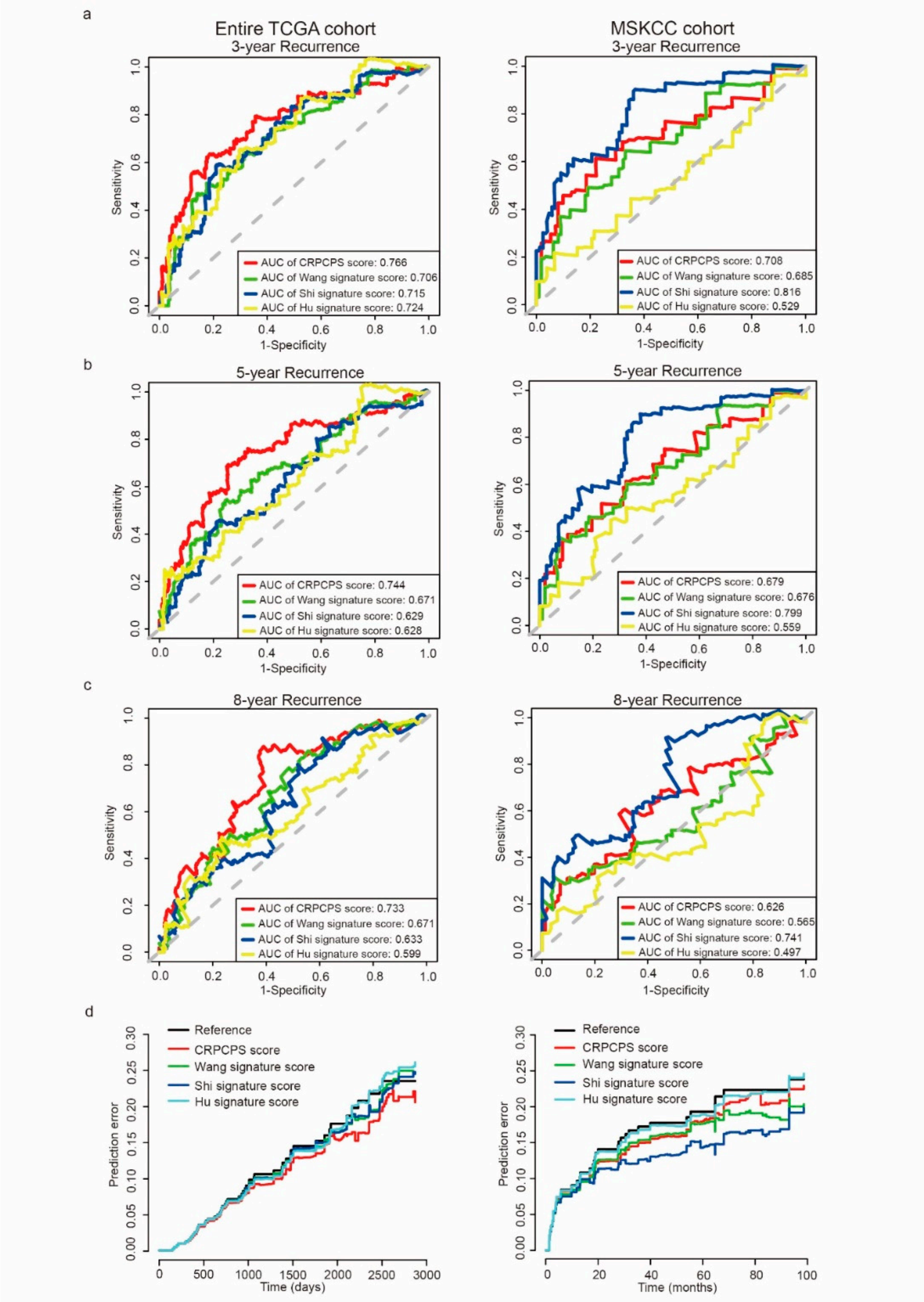

Figure S9. Performance of the CRPCPS and reported signatures in different cohorts. The area under the receiver operating characteristic (ROC) curves of the CRPCPS and reported signatures at 3- **(a)**, 5- **(b)**, and 8-year **(c)**, and the prediction error curves **(d)** in the entire TCGA cohort (left panel) and the MSKCC cohort (right panel).

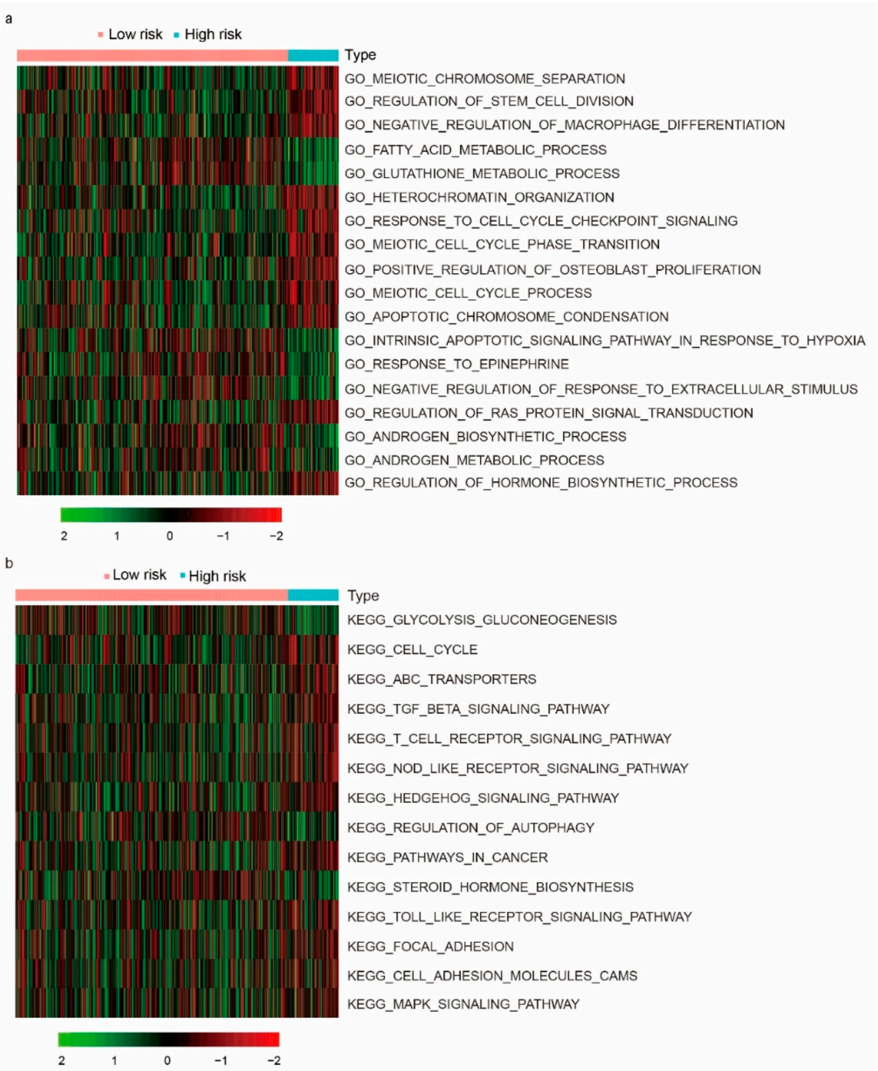

Figure S10. Functional enrichment analysis of the CRPCPS. GSVA identified the biological processes **(a)** and KEGG pathways **(b)** between patients with higher and lower CRPCPS scores.

Table S1. Characteristics of gene expression datasets used for the identification of differentially expressed genes.

Dataset ID	GPL ID	Platform	Number of samples	Reference
GSE6811	GPL4747	YN Human 36K (sets1-8)	13 CRPC; 10HSPC	Tamura K, et al. 2007 [1]
GSE2443	GPL96	Affymetrix Human Genome U133A Array	10 CRPC; 10 HSPC	Best CJ, et al. 2005 [2]
GSE28680	GPL10558	Illumina HumanHT-12 V4.0 expression beadchip	4 CRPC; 5 HSPC	Sharma NL, et al.2013 [3]
GSE70768	GPL10558	Illumina HumanHT-12 V4.0 expression beadchip	13 CRPC; 112 HSPC	Ross-Adams H, et al. 2015 [4]

GSE: Gene Expression Omnibus Series; GPL: Gene Expression Omnibus Platform; CRPC: castration-resistant prostate cancers; HSPC: hormone sensitive prostate cancer.

Table S2. Differentially expressed genes (DEGs) between localized CRPC and HSPC samples with |log2 (fold change)| > 0.5 and *p*-Value < 0.05 by RRA analysis. CRPC-associated genes newly identified in our study were marked in bold.

Gene Symbol	Entrez ID	Log2 (fold change)	*p*-Value
MSMB	4477	2.094	1.38×10^{-8}
DUSP1	1843	1.87	3.29×10^{-8}
CYR61	3491	1.665	9.81×10^{-8}
MT1X	**4501**	**1.341**	$\mathbf{1.21 \times 10^{-7}}$
TRPM8	79054	1.878	1.24×10^{-7}
ZFP36	7538	1.551	1.56×10^{-7}

SERPINA3	**12**	**1.603**	**7.38×10^{-7}**
TFF3	**7033**	**1.325**	**1.29×10^{-6}**
KLK3	354	1.347	1.42×10^{-6}
MYBPC1	4604	1.831	1.57×10^{-6}
CUX2	**23316**	**0.883**	**3.84×10^{-6}**
IER3	**8870**	**0.893**	**1.11×10^{-5}**
C1ORF115	**79762**	**1.016**	**1.31×10^{-5}**
SORD	**6652**	**1.089**	**1.60×10^{-5}**
ANPEP	290	1.539	1.82×10^{-5}
ATF3	467	1.278	2.89×10^{-5}
ANG	**283**	**0.882**	**2.93×10^{-5}**
GNMT	**27232**	**1.039**	**3.51×10^{-5}**
JUNB	3726	0.872	3.57×10^{-5}
FOSB	**2354**	**1.1**	**3.71×10^{-5}**
FOS	2353	1.677	4.61×10^{-5}
MT2A	**4502**	**0.905**	**6.35×10^{-5}**
CEBPD	**1052**	**0.895**	**6.52×10^{-5}**
ACPP	55	1.523	6.80×10^{-5}
NANS	54187	0.787	7.85×10^{-5}
DPP4	**1803**	**0.887**	**7.95×10^{-5}**
MT1H	4496	1.087	9.34×10^{-5}
BTG2	7832	0.791	1.04×10^{-4}
AMD1	**262**	**0.768**	**1.29×10^{-4}**
SLC45A3	**85414**	**1.308**	**1.31×10^{-4}**
MT1F	**4494**	**1.012**	**1.48×10^{-4}**
SLC15A2	6565	0.727	1.82×10^{-4}
EGR1	1958	1.43	1.89×10^{-4}
GDF15	**9518**	**0.876**	**2.05×10^{-4}**
XBP1	7494	0.877	2.36×10^{-4}
FEV	**54738**	**0.765**	**3.00×10^{-4}**
PAK1IP1	**55003**	**0.903**	**3.20×10^{-4}**
PTGDS	**5730**	**1.28**	**3.27×10^{-4}**
MT1M	4499	0.905	3.33×10^{-4}
SLC43A1	**8501**	**0.807**	**3.65×10^{-4}**
FMOD	**2331**	**0.738**	**3.73×10^{-4}**
AZGP1	**563**	**0.938**	**3.73×10^{-4}**
ANXA3	306	0.835	4.31×10^{-4}
C9ORF61	**9413**	**0.958**	**4.81×10^{-4}**
GLB1L2	**89944**	**1.029**	**4.92×10^{-4}**
NCAPD3	**23310**	**0.935**	**4.92×10^{-4}**
HGD	3081	0.973	5.08×10^{-4}
NBL1	4681	0.846	6.63×10^{-4}
GCAT	**23464**	**0.845**	**7.96×10^{-4}**
RPS14	6208	0.527	8.31×10^{-4}
GJB1	2705	1.099	8.93×10^{-4}
RPS25	**6230**	**0.61**	**8.98×10^{-4}**
LDLR	3949	0.651	9.19×10^{-4}
ABCC4	**10257**	**0.948**	**9.22×10^{-4}**
CSRP1	**1465**	**0.698**	**9.24×10^{-4}**
ID2	**3398**	**0.751**	**9.66×10^{-4}**
ALDH4A1	**8659**	**0.687**	**9.71×10^{-4}**
RAB27A	5873	0.9	9.88×10^{-4}
RPLP1	6176	0.567	1.03×10^{-3}
EGR2	**1959**	**0.801**	**1.05×10^{-3}**
HERPUD1	9709	0.745	1.05×10^{-3}
PAGE4	**9506**	**1.171**	**1.06×10^{-3}**
ALOX15B	**247**	**0.823**	**1.08×10^{-3}**
MT1E	**4493**	**0.755**	**1.09×10^{-3}**
P2RY5	10161	0.729	1.10×10^{-3}
GSTT1	**2952**	**0.692**	**1.17×10^{-3}**
SNORD3D	**780854**	**1.018**	**1.17×10^{-3}**
SLC14A1	**6563**	**0.762**	**1.20×10^{-3}**
DHCR24	**1718**	**0.671**	**1.31×10^{-3}**

FAM174B	**400451**	**0.616**	**1.31×10^{-3}**
KIF13B	**23303**	**0.515**	**1.45×10^{-3}**
RPL27A	6157	0.685	1.60×10^{-3}
GOLM1	**51280**	**0.689**	**1.63×10^{-3}**
RHOBTB3	**22836**	**0.569**	**1.78×10^{-3}**
KRT15	**3866**	**0.857**	**1.83×10^{-3}**
IL1R1	**3554**	**0.699**	**1.89×10^{-3}**
ASTN2	23245	0.801	1.97×10^{-3}
PARM1	**25849**	**0.783**	**1.98×10^{-3}**
JUN	3725	0.643	2.18×10^{-3}
KRT5	**3852**	**0.824**	**2.18×10^{-3}**
AIM1	**202**	**0.54**	**2.24×10^{-3}**
CNTNAP2	**26047**	**0.885**	**2.25×10^{-3}**
SLC1A5	**6510**	**0.644**	**2.28×10^{-3}**
F3	**2152**	**0.931**	**2.34×10^{-3}**
NPY	**4852**	**0.965**	**2.34×10^{-3}**
TNFSF10	**8743**	**0.672**	**2.35×10^{-3}**
EPHX2	2053	0.807	2.52×10^{-3}
GUCY1A3	**2982**	**0.749**	**2.67×10^{-3}**
GADD45G	10912	0.819	2.78×10^{-3}
ALDH1A3	**220**	**0.729**	**2.89×10^{-3}**
FLJ21511	**80157**	**0.522**	**3.05×10^{-3}**
SNORD3C	**780853**	**0.938**	**3.51×10^{-3}**
LOC124220	**124220**	**0.64**	**3.53×10^{-3}**
GSTM1	**2944**	**0.654**	**3.79×10^{-3}**
ARG2	**384**	**0.799**	**3.83×10^{-3}**
NR4A1	3164	1.027	4.47×10^{-3}
TMSB15A	**11013**	**0.619**	**4.51×10^{-3}**
PSMB8	**5696**	**0.625**	**4.66×10^{-3}**
SNORD3A	**780851**	**0.935**	**4.69×10^{-3}**
GZMA	**3001**	**0.533**	**4.85×10^{-3}**
UAP1	**6675**	**0.545**	**5.05×10^{-3}**
CORO1B	**57175**	**0.555**	**5.20×10^{-3}**
TXNRD2	**10587**	**0.568**	**5.35×10^{-3}**
BIK	**638**	**0.56**	**5.51×10^{-3}**
GREB1	9687	0.594	5.86×10^{-3}
EEF1G	**1937**	**0.719**	**5.96×10^{-3}**
SGSH	6448	0.515	6.19×10^{-3}
C6ORF160	**387066**	**0.592**	**6.26×10^{-3}**
CXCL2	**2920**	**0.638**	**6.60×10^{-3}**
SOX9	**6662**	**0.556**	**6.66×10^{-3}**
GMDS	**2762**	**0.571**	**6.72×10^{-3}**
RPS9	6203	0.601	6.83×10^{-3}
GPR25	**2848**	**0.532**	**7.03×10^{-3}**
ARSD	414	0.596	7.12×10^{-3}
REPS2	9185	0.637	7.18×10^{-3}
ALDH2	**217**	**0.561**	**7.42×10^{-3}**
MT1G	**4495**	**0.9**	**7.42×10^{-3}**
CECR6	**27439**	**0.664**	**7.48×10^{-3}**
S100A6	**6277**	**0.513**	**7.67×10^{-3}**
TACSTD2	4070	0.54	7.91×10^{-3}
LOC389787	**389787**	**0.519**	**7.98×10^{-3}**
CYP3A5	**1577**	**0.657**	**8.04×10^{-3}**
CNN1	**1264**	**0.571**	**8.10×10^{-3}**
NR4A2	**4929**	**0.566**	**8.16×10^{-3}**
EGR3	**1960**	**0.719**	**8.20×10^{-3}**
OR51E2	**81285**	**1.015**	**8.20×10^{-3}**
SCGB1A1	**7356**	**0.664**	**8.20×10^{-3}**
TPT1	**7178**	**0.606**	**8.29×10^{-3}**
CTGF	**1490**	**0.534**	**8.48×10^{-3}**
CIRBP	**1153**	**0.639**	**9.16×10^{-3}**
KCNN4	**3783**	**0.511**	**9.82×10^{-3}**
CYP27A1	1593	0.848	1.06×10^{-2}

CXADR	1525	0.543	1.07×10^{-2}
LRIG1	**26018**	**0.51**	$\mathbf{1.07 \times 10^{-2}}$
BACE2	**25825**	**0.536**	$\mathbf{1.14 \times 10^{-2}}$
STEAP4	**79689**	**0.548**	$\mathbf{1.16 \times 10^{-2}}$
EEF1A1	**1915**	**0.596**	$\mathbf{1.18 \times 10^{-2}}$
LCP1	3936	0.756	1.20×10^{-2}
ZFHX3	**463**	**0.628**	$\mathbf{1.20 \times 10^{-2}}$
RNF41	**10193**	**0.617**	$\mathbf{1.22 \times 10^{-2}}$
ST6GALNAC1	55808	0.809	1.29×10^{-2}
FAM107A	**11170**	**0.784**	$\mathbf{1.29 \times 10^{-2}}$
ACTG2	**72**	**0.692**	$\mathbf{1.29 \times 10^{-2}}$
SPRY2	10253	0.527	1.32×10^{-2}
SEC14L2	**23541**	**0.555**	$\mathbf{1.32 \times 10^{-2}}$
CDC42EP5	**148170**	**0.695**	$\mathbf{1.37 \times 10^{-2}}$
PDE4B	**5142**	**0.557**	$\mathbf{1.39 \times 10^{-2}}$
FGFR3	**2261**	**0.56**	$\mathbf{1.40 \times 10^{-2}}$
DBI	1622	0.509	1.56×10^{-2}
PTN	**5764**	**0.597**	$\mathbf{1.64 \times 10^{-2}}$
TARP	**445347**	**0.617**	$\mathbf{1.64 \times 10^{-2}}$
TIMP3	**7078**	**0.555**	$\mathbf{1.64 \times 10^{-2}}$
ETV4	**2118**	**0.583**	$\mathbf{1.76 \times 10^{-2}}$
C7	**730**	**0.69**	$\mathbf{1.76 \times 10^{-2}}$
C11ORF2	**738**	**0.527**	$\mathbf{1.81 \times 10^{-2}}$
LOC388654	**388654**	**0.502**	$\mathbf{1.83 \times 10^{-2}}$
INPP1	**3628**	**0.587**	$\mathbf{1.84 \times 10^{-2}}$
CRYM	1428	0.686	1.87×10^{-2}
TPSAB1	**7177**	**0.539**	$\mathbf{1.87 \times 10^{-2}}$
SH3BGRL2	83699	0.552	1.90×10^{-2}
CPE	**1363**	**0.56**	$\mathbf{2.02 \times 10^{-2}}$
LTF	**4057**	**0.568**	$\mathbf{2.11 \times 10^{-2}}$
BCAS3	54828	0.571	2.21×10^{-2}
IQGAP2	10788	0.598	2.22×10^{-2}
LAMB3	**3914**	**0.544**	$\mathbf{2.22 \times 10^{-2}}$
PIN1	**5300**	**0.517**	$\mathbf{2.22 \times 10^{-2}}$
NEFH	**4744**	**0.561**	$\mathbf{2.22 \times 10^{-2}}$
C12ORF57	**113246**	**0.591**	$\mathbf{2.24 \times 10^{-2}}$
RPS4X	6191	0.579	2.28×10^{-2}
KCNC2	**3747**	**0.543**	$\mathbf{2.34 \times 10^{-2}}$
KIAA1843	**84540**	**0.542**	$\mathbf{2.46 \times 10^{-2}}$
COL9A1	**1297**	**0.59**	$\mathbf{2.46 \times 10^{-2}}$
GADD45B	4616	0.61	2.52×10^{-2}
PDLIM5	**10611**	**0.502**	$\mathbf{2.53 \times 10^{-2}}$
PCA3	**50652**	**0.604**	$\mathbf{2.66 \times 10^{-2}}$
RPL9	**6133**	**0.513**	$\mathbf{2.75 \times 10^{-2}}$
VIPR1	7433	0.66	2.84×10^{-2}
CKB	**1152**	**0.59**	$\mathbf{2.91 \times 10^{-2}}$
ZNF750	**79755**	**0.581**	$\mathbf{3.04 \times 10^{-2}}$
SPOCK1	**6695**	**0.537**	$\mathbf{3.04 \times 10^{-2}}$
LOC642817	**642817**	**0.562**	$\mathbf{3.04 \times 10^{-2}}$
C19ORF18	**147685**	**0.521**	$\mathbf{3.16 \times 10^{-2}}$
LMO7	**4008**	**0.532**	$\mathbf{3.22 \times 10^{-2}}$
RPS23	**6228**	**0.522**	$\mathbf{3.29 \times 10^{-2}}$
TMEM30B	**161291**	**0.566**	$\mathbf{3.61 \times 10^{-2}}$
BTC	**685**	**0.516**	$\mathbf{3.62 \times 10^{-2}}$
LITAF	**9516**	**0.501**	$\mathbf{4.29 \times 10^{-2}}$
ACP2	53	0.604	4.35×10^{-2}
SMPD2	**6610**	**0.506**	$\mathbf{4.38 \times 10^{-2}}$
MEIS2	**4212**	**0.576**	$\mathbf{4.43 \times 10^{-2}}$
SNAP91	9892	0.564	4.44×10^{-2}
GLYATL1	**92292**	**0.591**	$\mathbf{4.67 \times 10^{-2}}$
AGTR1	**185**	**0.597**	$\mathbf{4.78 \times 10^{-2}}$
CREB3L4	**148327**	**0.571**	$\mathbf{4.78 \times 10^{-2}}$
COL5A2	1290	-0.86	1.68×10^{-5}

THBS2	**7058**	**-0.923**	**1.70×10^{-5}**
CDC20	**991**	**-0.964**	**2.12×10^{-5}**
COL1A1	**1277**	**-0.956**	**2.17×10^{-5}**
COL3A1	1281	-1.186	3.69×10^{-5}
THY1	**7070**	**-0.809**	**5.20×10^{-5}**
ISG15	**9636**	**-0.88**	**7.40×10^{-5}**
AIDA	**64853**	**-0.813**	**7.75×10^{-5}**
VWF	7450	-1.038	1.51×10^{-4}
COL1A2	1278	-1.16	1.65×10^{-4}
COL4A1	**1282**	**-0.883**	**1.84×10^{-4}**
TOP2A	**7153**	**-0.862**	**2.14×10^{-4}**
BCYRN1	**618**	**-0.765**	**3.08×10^{-4}**
WNT5A	**7474**	**-0.881**	**3.57×10^{-4}**
NUAK1	**9891**	**-0.671**	**3.80×10^{-4}**
COL5A1	**1289**	**-0.696**	**4.02×10^{-4}**
CAMK2N1	**55450**	**-0.731**	**4.49×10^{-4}**
CKS2	**1164**	**-0.845**	**5.09×10^{-4}**
COL6A3	**1293**	**-0.716**	**6.04×10^{-4}**
PRKAR2B	5577	-0.785	1.06×10^{-3}
ASPN	54829	-0.716	1.15×10^{-3}
TDO2	**6999**	**-0.925**	**1.17×10^{-3}**
LOC100132564	**100132564**	**-0.922**	**1.17×10^{-3}**
EEF1A2	**1917**	**-1.178**	**1.17×10^{-3}**
CEP55	**55165**	**-0.829**	**1.23×10^{-3}**
RCOR3	**55758**	**-0.556**	**1.26×10^{-3}**
STK3	6788	-0.784	1.28×10^{-3}
WDR67	**93594**	**-0.598**	**2.34×10^{-3}**
RN7SK	**125050**	**-0.892**	**2.34×10^{-3}**
ELTD1	**64123**	**-0.725**	**2.45×10^{-3}**
MTERFD1	**51001**	**-0.688**	**2.56×10^{-3}**
SDC2	6383	-0.56	2.57×10^{-3}
COL4A2	**1284**	**-0.661**	**2.60×10^{-3}**
NRP1	**8829**	**-0.742**	**2.71×10^{-3}**
FN1	2335	-0.662	2.89×10^{-3}
C4ORF15	**79441**	**-0.594**	**2.97×10^{-3}**
A2M	2	-0.541	3.05×10^{-3}
NPEPL1	79716	-0.572	3.24×10^{-3}
SESN3	143686	-0.597	3.51×10^{-3}
MIR1978	**100302173**	**-0.533**	**3.51×10^{-3}**
SULF1	**23213**	**-0.662**	**3.78×10^{-3}**
ECT2	**1894**	**-0.577**	**4.19×10^{-3}**
PNLIPRP1	5407	-0.59	4.69×10^{-3}
MYH2	**4620**	**-0.53**	**4.69×10^{-3}**
RNY5	**6090**	**-0.524**	**4.69×10^{-3}**
PPP1R16A	**84988**	**-0.653**	**5.51×10^{-3}**
CDKN2C	1031	-0.55	5.67×10^{-3}
PARP1	**142**	**-0.509**	**5.72×10^{-3}**
E2F3	**1871**	**-0.519**	**5.85×10^{-3}**
EIF2C2	27161	-0.767	5.86×10^{-3}
GRP	**2922**	**-0.51**	**5.86×10^{-3}**
LOC100008588	**100008588**	**-0.512**	**5.86×10^{-3}**
PUF60	**22827**	**-0.543**	**5.91×10^{-3}**
C20ORF20	**55257**	**-0.516**	**5.93×10^{-3}**
HOPX	**84525**	**-0.727**	**6.04×10^{-3}**
XPO1	7514	-0.501	6.22×10^{-3}
VIM	**7431**	**-0.634**	**7.03×10^{-3}**
FLJ38717	**401261**	**-0.501**	**7.03×10^{-3}**
GP2	**2813**	**-0.635**	**7.03×10^{-3}**
TOPBP1	11073	-0.507	7.76×10^{-3}
ENO2	**2026**	**-0.563**	**8.04×10^{-3}**
DTL	51514	-0.589	8.2×10^{-3}
PLOD3	**8985**	**-0.532**	**8.31×10^{-3}**
RGS5	**8490**	**-0.769**	**8.36×10^{-3}**

CXCR7	**57007**	**-0.632**	**8.48×10^{-3}**
PCDH17	**27253**	**-0.676**	**8.55×10^{-3}**
C1ORF64	**149563**	**-0.547**	**9.37×10^{-3}**
SPARC	6678	-0.688	9.75×10^{-3}
PTPRU	10076	-0.552	1.05×10^{-2}
MMD	**23531**	**-0.579**	**1.10×10^{-2}**
ZFP41	286128	-0.565	1.17×10^{-2}
SQLE	**6713**	**-0.659**	**1.19×10^{-2}**
SHMT2	**6472**	**-0.63**	**1.27×10^{-2}**
CYP4F8	**11283**	**-0.549**	**1.29×10^{-2}**
IQGAP3	**128239**	**-0.541**	**1.40×10^{-2}**
NPTX2	**4885**	**-0.676**	**1.52×10^{-2}**
LAMB1	3912	-0.507	1.54×10^{-2}
LBH	**81606**	**-0.518**	**1.64×10^{-2}**
CDH11	1009	-0.62	1.84×10^{-2}
LAMC1	**3915**	**-0.531**	**1.94×10^{-2}**
IFI44L	**10964**	**-0.531**	**2.06×10^{-2}**
PNCK	**139728**	**-0.634**	**2.11×10^{-2}**
TIGD5	**84948**	**-0.529**	**2.17×10^{-2}**
CORO1A	**11151**	**-0.527**	**2.22×10^{-2}**
SMC4	10051	-0.65	2.26×10^{-2}
PRDX4	**10549**	**-0.518**	**2.46×10^{-2}**
COMP	**1311**	**-0.501**	**2.69×10^{-2}**
SAMSN1	**64092**	**-0.568**	**3.16×10^{-2}**
RNASE1	**6035**	**-0.539**	**3.31×10^{-2}**
PLOD1	**5351**	**-0.509**	**3.75×10^{-2}**
KL	**9365**	**-0.516**	**4.05×10^{-2}**
GPR172A	**79581**	**-0.562**	**4.10×10^{-2}**
KIAA0101	**9768**	**-0.54**	**4.78×10^{-2}**

Table S3. List of the 24 genes that were significantly associated with recurrence-free survival (RFS) in the TCGA training cohort (p-Value < 0.05), as analyzed by the univariate Cox regression analysis.

Gene	coef	exp (coef)	se (coef)	z value	*p*-Value
HOPX	0.783	2.187	0.356	2.195	0.028
NPEPL1	0.942	2.564	0.364	2.585	0.01
RGS5	0.85	2.34	0.366	2.326	0.02
PARM1	−0.838	0.432	0.365	−2.298	0.022
SLC45A3	−0.941	0.39	0.374	−2.514	0.012
VWF	0.738	2.091	0.365	2.022	0.043
NRP1	1.08	2.945	0.388	2.786	0.005
TMEM30B	−0.763	0.466	0.357	−2.138	0.032
LMO7	−0.837	0.433	0.364	−2.299	0.022
RPL27A	−0.938	0.391	0.364	−2.577	0.01
ALDH2	−0.877	0.416	0.388	−2.256	0.024
EPHX2	−0.929	0.395	0.388	−2.398	0.016
PTN	−0.748	0.473	0.376	−1.989	0.047
REPS2	−0.712	0.491	0.356	−1.997	0.046
PCDH17	0.722	2.058	0.365	1.98	0.048
NUAK1	1.059	2.884	0.378	2.801	0.005
CDH11	0.787	2.197	0.364	2.16	0.031
GOLM1	−1.042	0.353	0.365	−2.852	0.004
XPO1	0.884	2.419	0.369	2.394	0.017
PLOD3	0.781	2.185	0.364	2.145	0.032
DPP4	−0.866	0.421	0.375	−2.307	0.021
TPT1	−1.038	0.354	0.375	−2.771	0.006
ZG16B	−0.814	0.443	0.364	−2.235	0.025
C12orf57	−0.982	0.374	0.389	−2.523	0.012

Table S4. Characteristics of the three CRPCPS-derived genes and their coefficients used for predicting CRPC occurrence.

Gene	Coefficients	SE	z Value	*p*-Value
NPEPL1	9.333	1.896	4.923	< 0.0001
LMO7	−9.577	2.358	−4.062	< 0.0001
TPT1	−8.043	1.904	−4.224	< 0.0001

References

1. Tamura, K.; Furihata, M.; Tsunoda, T.; Ashida, S.; Takata, R.; Obara, W.; Yoshioka, H.; Daigo, Y.; Nasu, Y.; Kumon, H.; et al. Molecular features of hormone-refractory prostate cancer cells by genome-wide gene expression profiles. *Cancer Res.* **2007**, *67*, 5117–5125, doi:10.1158/0008-5472.CAN-06-4040.
2. Best, C.J.; Gillespie, J.W.; Yi, Y.; Chandramouli, G.V.; Perlmutter, M.A.; Gathright, Y.; Erickson, H.S.; Georgevich, L.; Tangrea, M.A.; Duray, P.H.; et al. Molecular alterations in primary prostate cancer after androgen ablation therapy. *Clin Cancer Res.* **2005**, *11*, 6823–6834, doi:10.1158/1078-0432.CCR-05-0585.
3. Sharma, N.L.; Massie, C.E.; Ramos-Montoya, A.; Zecchini, V.; Scott, H.E.; Lamb, A.D.; MacArthur, S.; Stark, R.; Warren, A.Y.; Mills, I.G.; et al. The androgen receptor induces a distinct transcriptional program in castration-resistant prostate cancer in man. *Cancer Cell* **2013**, *23*, 35–47, doi:10.1016/j.ccr.2012.11.010.
4. Ross-Adams, H.; Lamb, A.D.; Dunning, M.J.; Halim, S.; Lindberg, J.; Massie, C.M.; Egevad, L.A.; Russell, R.; Ramos-Montoya, A.; Vowler, S.L.; et al. Integration of copy number and transcriptomics provides risk stratification in prostate cancer: A discovery and validation cohort study. *EBioMedicine* **2015**, *2*, 1133–1144, doi:10.1016/j.ebiom.2015.07.017.
